# Age-related differences of genetic susceptibility to patients with acute lymphoblastic leukemia

**DOI:** 10.18632/aging.202903

**Published:** 2021-04-23

**Authors:** Qing Hao, Minyuan Cao, Chunlan Zhang, Dandan Yin, Yuelan Wang, Yuanxin Ye, Shan Zhao, Yunfan Yang, Ke-Ling Chen, Binwu Ying, Lanlan Wang, Yiguan Zhang, Caigang Xu, Yiping Zhu, Yu Wu, Ju Gao, Jun-Ning Zhao, Yan Zhang, Xiaoxi Lu

**Affiliations:** 1 Department of Thoracic Oncology, Cancer Center, West China Hospital, Sichuan University, Chengdu, China; 2 College of Pharmaceutical Sciences, Southwest Medical University, Luzhou, China; 3 Sichuan Center for Translational Medicine of Traditional Chinese Medicine, Institute of Translational Pharmacology, Sichuan Academy of Chinese Medicine Sciences, Chengdu, China; 4 Department of Laboratory Medicine, West China Hospital, Sichuan University, Chengdu, China; 5 Department of Hematology, West China Hospital, Sichuan University, Chengdu, China; 6 Digestive Surgery Institute, West China Hospital, Sichuan University, Chengdu, China; 7 Department of Hematology/Oncology, West China Second Hospital, Sichuan University, Chengdu, China

**Keywords:** acute lymphoblastic leukemia, genetic susceptibility, genome-wide association study, age specific

## Abstract

Inherited predispositions to acute lymphoblastic leukemia have been well investigated in pediatric patients, but studies on adults, particularly Chinese patients, are limited. In this study, we conducted a genome-wide association study in 466 all-age Chinese patients with Acute lymphoblastic leukemia (ALL) and 1,466 non-ALL controls to estimate the impact of age on ALL susceptibility in the Chinese population. Among the 17 reported loci, 8 have been validated in pediatric and 1 in adult patients. The strongest association signal was identified at *ARID5B* locus and gradually decreased with age, while the signal at *GATA3* exhibited the opposite trend and significantly impact on adult patients. With genome-wide approaches, germline variants at 2q14.3 rank as the top inherited predisposition to adult patients (e.g., rs73956024, *P* = 4.3 × 10^-5^) and separate the genetic risk of pediatric vs. adult patients (*P* = 3.6 × 10^-6^), whereas variants at 15q25.3 (e.g., rs11638062) have a similar impact on patients in different age groups (overall *P* = 2.9 × 10^-7^). Our analysis highlights the impact of age on genetic susceptibility to ALL in Chinese patients.

## INTRODUCTION

Acute lymphoblastic leukemia (ALL) is a deadly malignancy for both children and adults, mainly observed in children aged between 2 and 5 years old [1]. Although over 80% of pediatric patients can experience 5-year survival after conventional chemotherapeutics [2], the survival rate is still low in adult patients [3, 4]. Genomically, higher prevalence of ALL genetic subtypes with poor prognosis (e.g., BCR-ABL) and lower subtypes with favorable outcomes (e.g., hyperdiploidy) were presented in adults, therefore, it can partially explain the different survival rate between pediatric and adult patients [4, 5]. However, the genetic determinant for age-specific leukemogenesis is still controversial.

On the other hand, germline variants can strongly influence both susceptibility and treatment outcomes of ALL. Through genome-wide association studies (GWAS), multiple inherited predispositions to ALL in pediatric patients have been identified, including single nucleotide polymorphisms (SNPs) at loci of *ARID5B*, *IKZF1*, *GATA3,* etc. [6–20]. Association of some SNPs are greatly impacted by clinical characteristics (e.g., age, ethnicity, and subtypes) [8, 13–17, 19, 21], which can be validated by a series of replication studies in independent patient cohorts [22–29]. Recently, with the GWASs performed in specific ethnicity and meta-analysis with a large sample size conducted based on GWASs, four novel loci were identified including Hispanic specific locus (i.e., rs2836365 in *ERG*) [13], and hyperdiploid subtype specific locus (e.g., rs210143 in *BAK1*) [17]. Therefore, a total of 17 pediatric ALL susceptibility loci with genome-wide significance have been reported after several years of investigations. However, studies on inherited predispositions to ALL risk for Chinese patients are limited [30], particularly validations for the recently reported loci. Besides, there are relatively fewer studies on adult patients [31], especially on adolescents and young adults [10]. We thus conducted GWAS in all-age Chinese patients with ALL, to evaluate the reported GWAS signals and screen novel genetic variants, which have age-related different effects on ALL.

## MATERIALS AND METHODS

### Subject and genotyping

Peripheral blood was obtained from 1,466 non-ALL controls, as well as 466 B-linage ALL patients (381 childhood [0-14 yrs] and 85 adult patients [14-68 yrs] who were treated with standard protocol in West China Hospital of West China Second Hospital (e.g., CCGC-ALL2015, registered in http://www.chictr.org.cn/ with ID: ChiCTR-IPR-14005706). Clinical information was obtained from the record system at our hospitals, including gender, age at diagnosis, and molecular subtypes. Fusion-based molecular subtypes were determined by fluorescence *in situ* hybridization.

A total of 811,852 SNPs were genotyped with Precision Medicine Research Array (ThermoFisher) and filtered based on minor allele frequency, call-rate, Hardy-Weinberg equilibrium, etc., according to previous standard steps [8]. Subsequently, imputation was conducted by well-established methods (i.e., Michigan Imputation Server [32]) with the filtered SNPs. After setting *r^2^* = 0.5 as a cutoff threshold, 9,466,286 SNPs were finally used for subsequent association analysis.

### Statistical analysis

Four GWAS approaches were conducted: all patients *vs.* non-ALL control, pediatric patients (< 14yrs) *vs.* non-ALL controls, adult patients *vs.* non-ALL controls, and pediatric patients *vs.* adult patients. For the reported loci, subtype-specific associations were evaluated. For statistical analysis, the association of SNP genotypes with the indicated phenotypes (e.g., ALL susceptibility of all-age patients) were estimated by comparing the genotype frequency between ALL cases and non-ALL controls, or different age groups with logistic regression model after adjusting for gender and the top three principal components. *P* value, odds ratio (OR) and 95% confidence interval (95% CI) was estimated by using PLINK (version 1.90) [33].

## RESULTS

**Figure 1:**
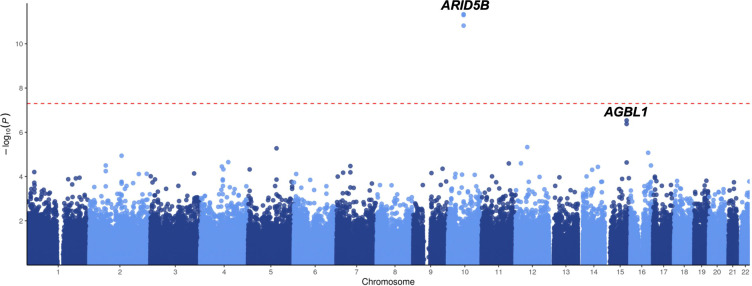
**GWAS results of ALL susceptibility in all-age Chinese patients.** Association between SNPs and ALL was evaluated in 466 ALL cases and 1,466 non-ALL controls. P value was estimated by logistic regression test and -log_10_ P (*y*-axis) were plotted against the respectively chromosomal position of each SNP (*x*-axis). Only genotyped but not imputed SNPs were illustrated.

To investigate the inherited predispositions to ALL in the Chinese population, GWAS was performed with all imputed SNPs after stringently filtering. A total of 1,466 non-ALL controls and 466 B-linage ALL patients were included in this study with baseline characteristics illustrated in Table 1, including 381 pediatric (< 14 yrs] and 85 adult patients (≥ 14yrs). For childhood ALL (< 14yrs), only one locus (i.e., *ARID5B*) reached genome-wide significance (*P* < 5 × 10^-8^) (Figure 1), suggesting no novel strong genetic predisposition to ALL in the Chinese population with current sample size. Subsequently, we retrieved the association results for all 21 SNPs at 17 reported loci from ALL patients and non-ALL controls. In pediatric patients, a total of 7 SNPs at 6 loci were significantly associated with ALL susceptibility regardless of molecular subtypes, including SNPs at *ARID5B*, *IKZF1*, *BMI-PIP4K2A*, *CEBPE*, *CDKN2B-AS1*, and *BAK1* (Table 2). The top signal at *ARID5B* was rs7090445 (*P* = 2.7 × 10^-14^, Odds ratio [OR] = 1.96, [95% CI: 1.60-2.24]), which was reported as the potential causal variant for *ARID5B* locus. rs11770117 (*P* = 0.03, OR = 1.27 [1.01-1.62]) at *IKZF1* locus exhibited independent association with ALL susceptibility even after adjusting for the top signal at *IKZF1* locus (i.e., rs11978267, *P* = 0.003, OR = 1.40 [1.12-1.75]) with *P*_adjust_ = 0.04, which is consistent with previous observation in Hispanics [8]. Additionally, rs4266962 (*P* = 0.01, OR = 1.64 [1.11-2.43]) also exhibited significance independent of rs7088318 at *BMI-PIP4K2A* locus. Among three recently identified loci with a large sample size of the Caucasian population, only rs210143 (*P* = 0.007, OR = 1.41 [1.10-1.81]) at *BAK1* locus can be validated in our Chinese patients (Table 2), indicating ethnicity-specific and shared mechanism of leukemogenesis. However, in adult patients, only SNPs at *GATA3* locus (rs3824662) exhibited significant association with ALL susceptibility (*P* = 0.0005, OR = 1.79 [1.29-2.50]) (Table 2), suggesting the different genetic basis of adult from pediatric patients.

**Table 1:** Clinical characteristics of all-age ALL patients.

**Clinical features**	**Childhood (N=381)**	**Adult (N=85)**
Age at diagnosis (years); median ± *SD*	4.7± 3.1	32± 12.8
**Gender**		
Male, *N* (%)	210 (55.1%)	48 (56.5%)
Female, *N* (%)	171 (44.9%)	37 (43.5%)
WBC (× 10^9^/L); median ± *SD*	10.2 ± 84.8	20.63 ± 99.0
**Molecular subtype**		
*ETV6-RUNX1*	76	1
*TCF3-PBX1*	18	0
*BCR-ABL* (Ph^+^)	16	46
Hyperdiploid*	31	0

*Hyperdiploid subtype was not determined in 161 out of 466 patients, because it is not routinely tested before 2016 in pediatric ALL and never tested in adults.

**Table 2:** Association of the GWAS hits with ALL susceptibility in Chinese patients.

**SNP ID**	Position*	**Genes**	**Childhood**						**Adult(N = 85)**		**Age difference**
			**all (N = 381)**		***ETV6-RUNX1* (N = 77)**		**no fusion B-ALL (N = 219)**				
			**P value**	**OR (95% CI)**	**P value**	**OR (95% CI)**	**P value**	**OR (95% CI)**	**P value**	**OR (95% CI)**	**P value**
rs17481869	2:146124454	*ZEB2*	1	NA	1	NA	1	NA	1	NA	1
rs886285	5:131765206	*C5orf56*	0.85	1.02 (0.86-1.21)	0.84	1.04 (0.74-1.45)	0.29	1.12 (0.91-1.37)	0.29	1.20 (0.86-1.66)	0.5
rs210143	6:33546930	***BAK1***	**0.007**	1.41 (1.10-1.81)	0.41	1.22 (0.76-1.94)	**0.017**	1.45 (1.07-1.96)	0.15	1.33 (0.90-1.96)	**0.007**
rs11978267	7:50466304	***IKZF1***	**0.003**	1.40 (1.12-1.75)	0.09	1.45 (0.95-2.21)	**0.006**	1.45 (1.11-1.88)	0.55	1.14 (0.73-1.78)	0.41
rs11770117	7:50473763	***IKZF1***	**0.03**	1.27 (1.01-1.62)	0.39	1.23 (0.77-1.97)	**0.01**	1.51 (1.10-2.05)	0.34	1.25 (0.79-1.96)	0.89
rs28665337	8:130194104	*MYC*	0.27	1.46 (0.74-2.90)	0.43	1.60 (0.50-5.18)	0.35	1.29 (0.76-2.19)	0.54	1.39 (0.49-3.95)	0.32
rs3731249	9:21970916	*CDKN2A*	1	NA	1	NA	1	NA	1	NA	1
rs10811641	9:22014137	***CDKN2B-AS1***	**0.013**	1.24 (1.04-1.47)	**0.024**	1.45 (1.05-2.01)	0.13	1.17 (0.96-1.42)	0.7	1.07 (0.76-1.49)	0.31
rs17756311	9:22053895	*CDKN2A*	0.4541	1.36 (0.61-3.06)	0.42	1.77 (0.45-7.01)	0.34	1.45 (0.68-3.10)	0.37	1.90 (0.47-7.69)	0.76
rs76925697	9:83747371	*TLE1*	1	NA	1	NA	1	NA	1	NA	1
rs3824662	10:8104208	***GATA3***	0.1526	1.14 (0.95-1.35)	0.73	1.06 (0.76-1.49)	**0.032**	1.25 (1.02-1.54)	**0.0005**	1.79 (1.29-2.50)	**0.05**
rs4266962	10:22341574	***BMI-PIP4K2A***	**0.013**	1.64 (1.11-2.43)	0.98	1.01 (0.52-1.95)	**0.013**	1.98 (1.16-3.38)	0.89	1.05 (0.54-2.01)	0.55
rs7088318	10:22852948	***BMI-PIP4K2A***	0.21	1.11 (0.94-1.31)	0.46	1.13 (0.82-1.57)	**0.05**	1.21 (1.00-1.49)	0.12	1.61 (0.88-2.95)	0.8
rs7090445	10:63723577	***ARID5B***	**2.7×10^-14^**	1.96 (1.60-2.24)	**0.002**	1.69 (1.21-2.34)	**8.2×10^-13^**	2.29 (1.85-2.82)	0.36	1.16 (0.84-1.60)	**0.003**
rs35837782	10:126293309	***LHPP***	0.09	1.17 (0.98-1.40)	0.56	1.10 (0.79-1.55)	**0.044**	1.23 (1.00-1.51)	0.67	1.08 (0.77-1.51)	0.33
rs4762284	12:96612762	*ELK3*	0.72	1.03 (0.87-1.23)	0.43	1.15 (0.81-1.61)	0.35	1.10 (0.90-1.36)	0.71	1.07 (0.75-1.52)	0.61
rs2239630	14:23589349	***CEBPE***	**0.007**	1.29 (1.07-1.54)	**0.016**	1.53 (1.08-2.15)	**0.013**	1.30 (1.06-1.60)	0.21	1.26 (0.88-1.80)	**0.033**
rs1121404	16:79089869	*WWOX*	0.34	1.13 (0.88-1.45)	0.88	1.03 (0.72-1.48)	0.7	1.04 (0.83-1.31)	0.28	1.34 (0.78-2.31)	0.56
rs2290400	17:38066240	*IKZF3*	0.51	1.07 (0.88-1.29)	0.84	1.04 (0.72-1.51)	0.64	1.06 (0.84-1.32)	0.6	1.10 (0.77-1.56)	0.79
rs10853104	17:47092076	*IGF2BP1*	0.53	1.08 (0.84-1.39)	0.5	1.17 (0.74-1.86)	0.96	1.01 (0.75-1.36)	0.81	1.08 (0.59-1.95)	0.3
rs2836365	21:39768274	***ERG***	0.069	1.18 (0.99-1.41)	0.39	1.17 (0.82-1.67)	**0.022**	1.29 (1.04-1.61)	0.94	1.02 (0.70-1.47)	0.88

*Chromosomal locations are based on hg19. Bold genes indicate that SNPs at this locus exhibit significance with ALL susceptibility at least in one association test. P values and ORs were estimated by the logistic regression test, and bold P values indicate P<0.05. OR, odds ration; CI, confidence interval.

**Figure 2:**
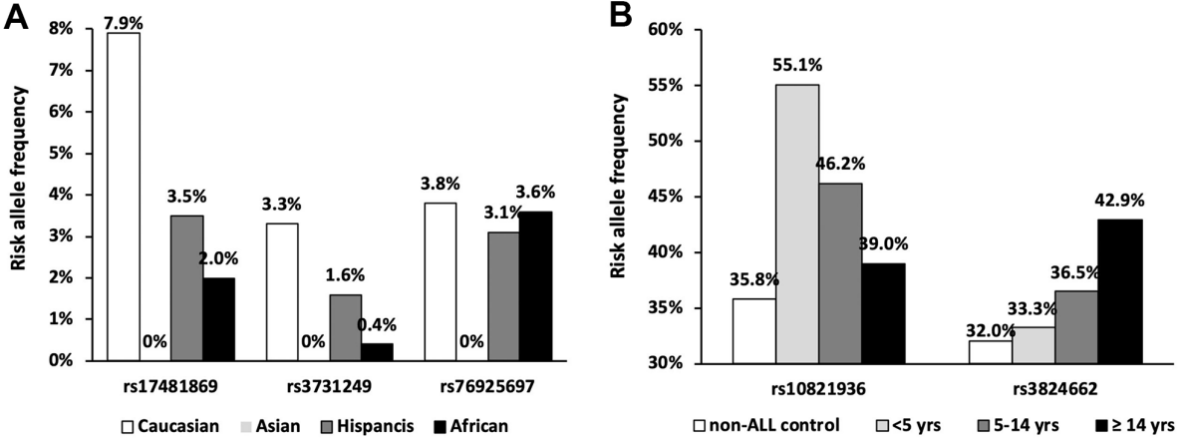
**Frequency of the ethnic and age specific loci.** (**A**) Risk allele frequencies were illustrated in SNPs with absent variant allele in Chinese population according to gnomAD database; (**B**) Risk allele frequencies of SNPs at *ARID5B* and *GATA3* loci in patients with different age group.

Since the significance of some GWAS signals is greatly impacted by clinical features, we thus estimated their associations with ALL susceptibility in Chinese patients considering ethnicity, age, and molecular subtype. The best example for ethnic specificity is the causal missense variant (i.e., rs3731249) in *CDKN2A*. Risk allele frequency (RAF) of rs3731249 is absent (0%) in our cohort, which is consistent with that in a public database (i.e., 0% in East Asian *vs.* 3.3% in Caucasians according to gnomAD [34]) (Figure 2A), and thus perfectly explain the racial difference at this locus. The insignificance of rs17481869 at 2q22.3 locus and rs76925697 at 9q21.31 locus can also be explained by ethnicity specific risk allele frequency. In pediatric ALL patients, the novel Hispanic-specific ALL risk signal at *ERG* locus exhibited marginally significant association with ALL susceptibility in Chinese patients (*P* = 0.07, OR = 1.18 [0.99-1.41]), and reached statistical significance in patients without common fusion (*P* = 0.02, OR = 1.29 [1.04-1.61]), which has been validated in previous study [28]. However, rs1121404 (*P* = 0.34, OR = 1.13 [0.88-1.45]) at *WWOW* identified in Chinese specific GWAS cannot be validated in our cohort. Association analysis was next performed in different genetic subtypes. Although association of rs7088318 at *BMI*-*PIP4K2A* locus with ALL susceptibility did not reach statistical significance in the whole patient cohort (*P* = 0.21, OR = 1.11 [0.94-1.31]), risk allele of this SNP was enriched in B-ALL patients with no common fusions (*P* = 0.05, OR = 1.21 [1.00-1.49]). Similarly, rs35837782 at hyperdiploid-specific *LHPP* locus exhibited marginally significant association with ALL susceptibility in the whole cohort (*P* = 0.09, OR = 1.17 [0.98-1.40]), but achieved statistical significance in B-ALL patient with no common fusions (*P* = 0.04, OR = 1.23 [1.00-1.51]), which is also observed for rs2836365 in *ERG* (*P* = 0.02, OR = 1.29 [1.04-1.61]). Consistent with the observation in Hispanics [13], an enriched risk allele of rs2836365 was observed in the *TCF3-PBX1* subtype at rs2836365. In contrast, *ETV6-RUNX1* subtype specific SNPs rs10853104 at *IGF2BP1* locus (*P* = 0.53, OR = 1.08 [0.84-1.39]) at 17q21.32 cannot be validated in our cohort even after considering different subtypes.

Next, we evaluated the impact of age on genetic predisposition. Among all the reported GWAS loci, four were significantly associated with age at diagnosis, namely *ARID5B*, *GATA3*, *BAK1* and *CEBPE* (Table 2). Particularly, all signals lost their association with ALL susceptibility in adults except rs3824662 at *GATA3* locus. As an example, the impact of rs10821936 at *ARID5B* locus gradually decreased from <5 yrs (RAF= 0.55, *P* = 2.6 × 10^-12^, OR = 2.27 [1.29-2.09]), 5-14 yrs, (RAF = 0.46, *P* = 6.4 × 10^-5^, OR = 1.63 [1.29-2.09]), to ≥14 yrs (RAF = 0.39, *P* = 0.24, OR = 1.23 [0.87-1.72]). Contrastly, rs3824662 at *GATA3* locus exhibited the opposite trend, with increased impact from <5 yrs (RAF = 0.33, *P* = 0.43, OR = 1.09 [0.87-1.36-2.09]), 5-14 yrs (RAF = 0.37, *P* = 0.03, OR = 1.31 [1.03-1.67]), and ≥14 yrs (RAF = 0.43, *P* = 0.0005, OR = 1.79 [1.29-2.50]) Figure 2B. To investigate the impact of age on ALL susceptibility, we compared allele frequencies of each SNP between pediatric and adult patients with GWAS approach by using logistic regression model (λ=1.03 for the quantile-quantile plot). Although no locus reached genome-wide significance, one novel locus was identified at 2q14.3 with the top signal of rs73956024 (*P* = 3.6 × 10^-6^) (Figure 3A and Table 3). Additionally, to identify novel ALL susceptibility loci in different age of Chinese patients, we also preformed GWASs in pediatric and adult patients separately. Only *ARID5B* locus reached genome-wide significance in pediatric patients, but none for adult patients, probably because of the small sample size. Interestingly, signals at 2q14.3 locus, which was described above, also ranked the top in adult (e.g., rs73956024, *P* = 4.5 × 10^-5^, OR = 2.31 [1.55-3.45]) but not significant in pediatric patients (Figure 3B and Table 3), with RAF of 0.08 in pediatric patients compared with 0.21 and 0.10 in adult patients and non-ALL controls, respectively. Moreover, rs11638062 at 15q25.3 has impact on ALL susceptibility in both pediatric (*P* = 4.2 × 10^-5^_,_ OR = 1.67 [1.31-2.14]) and adult patients (*P* = 4.7 × 10^-6^_,_ OR = 2.53 [1.70-3.77]), and the overall susceptibility of this locus reached marginally genome-wide significance for all-age patients (*P* = 2.9 × 10^-7^, OR = 1.80 [1.44-2.25]) (Figures 1, 3C and Table 3). However, validation in independent patient cohorts is needed due to the small sample size of our study.

**Figure 3a:**
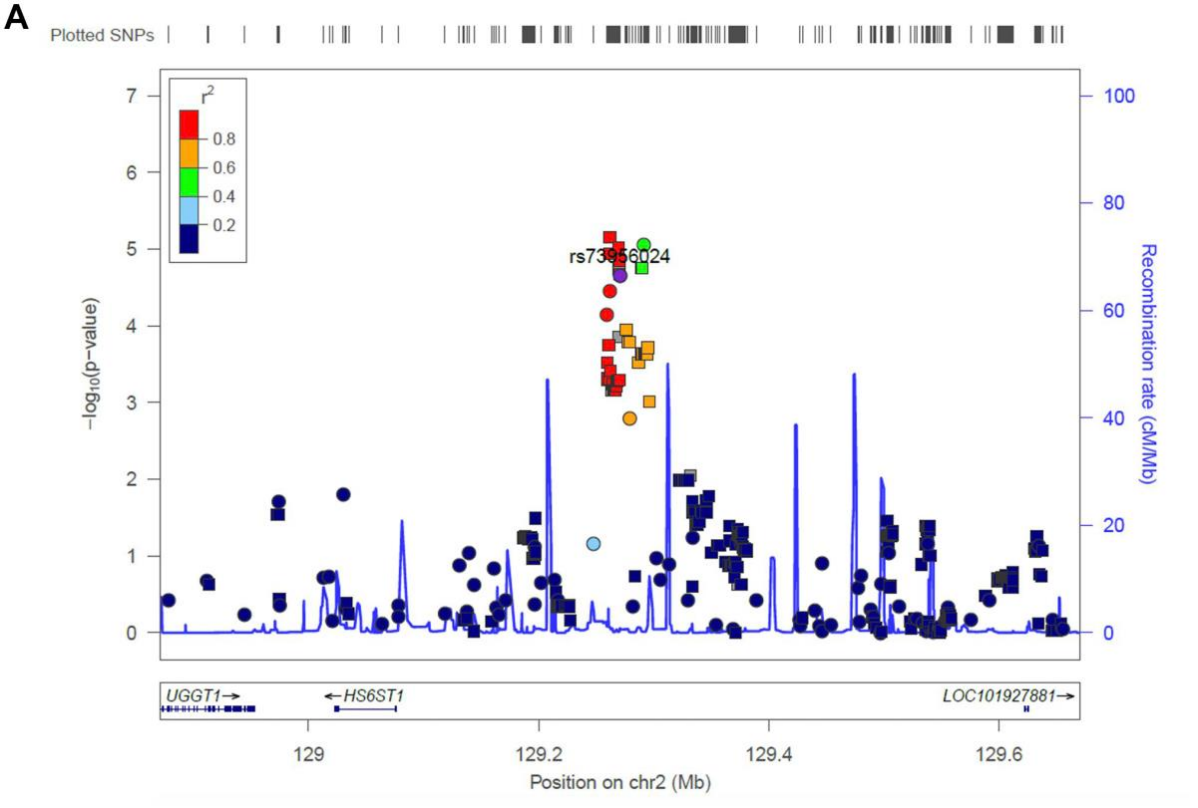
**Regional association plot of the novel loci.** Association of SNPs at 15q25.3 with ALL susceptibility in adult (**A**) and childhood (**B**). (**C**) Association of SNPs at 2q14.3 with ALL susceptibility in all-age patients. Genotyped and imputed SNPs were labeled in circles and squares, respectively.

**Table 3:** Association status of the novel loci.

**SNP ID**	Position*	**Genes**	**Childhood**		**Adult**		**All-age patients**		**Adults *vs.* Children**	
			**P value**	**OR (95% CI)**	**P value**	**OR (95% CI)**	**P value**	**OR (95% CI)**	**P value**	**OR (95% CI)**
rs11638062	15:86620033	*AGBL1*	**4.2×10^-5^**	1.67 (1.31-2.14)	**4.7×10^-6^**	2.53 (1.70-3.77)	**2.9×10^-7^**	1.80 (1.44-2.25)	0.06	1.53 (0.98-2.38)
rs16977928	15:86684441	*AGBL1*	**0.0003**	1.80 (1.31-2.48)	**0.023**	1.94 (1.09-3.43)	**2.3×10^-5^**	1.90 (1.41-2.56)	0.97	1.01 (0.55-1.85)
rs73956024	2:128513332	*HS6ST1*	0.13	0.81 (0.64-1.05)	**4.3×10^-5^**	2.31 (1.55-3.45)	0.96	1.01 (0.78-1.30)	**3.6×10^-6^**	3.11 (1.93-5.04)

*Chromosomal locations are based on hg19. Bold genes indicate that SNPs at this locus exhibit significance with ALL susceptibility at least in one association test. P values and ORs were estimated by the logistic regression test, and bold P values indicate *P* < 0.05; OR, odds ration; CI, confidence interval.

**Figure 3b:**
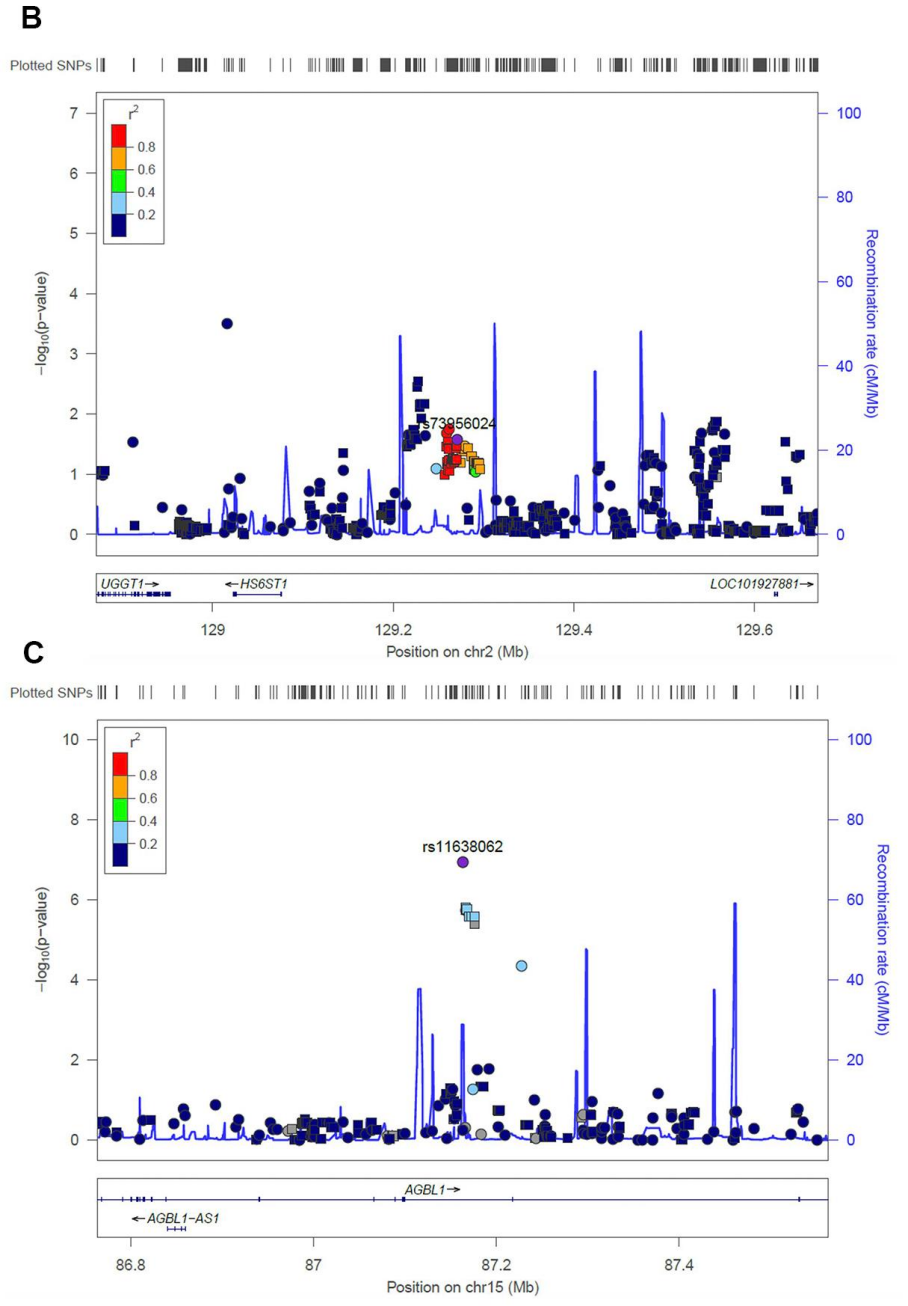
**Regional association plot of the novel loci.** Association of SNPs at 15q25.3 with ALL susceptibility in adult (**A**) and childhood (**B**). (**C**) Association of SNPs at 2q14.3 with ALL susceptibility in all-age patients. Genotyped and imputed SNPs were labeled in circles and squares, respectively.

## DISCUSSION

Most inherited dispositions to ALL have been revealed in Caucasians through genome-wide approaches but limited in Chinese patients. Although subsequent validations have been proceeded for the early identified loci (e.g., *ARID5B*), the impact of the novel loci identified recently with a large sample size has not been evaluated, particularly those ages-, ethnicity- and subtype-specific variants. In this study, we systematically investigated the reported GWAS signals for ALL susceptibly in all-age Chinese patients, and estimated the impact of clinical features, particularly age at diagnosis on ALL susceptibility. A total of 11 SNPs located at 9 out of 17 loci can be validated in Chinese patients in the whole cohort or specific subgroup. The inconsistency is probably due to the racial difference of inherited predispositions to ALL and the small sample size (particularly for some molecular subtypes). Except for the absence of missense variants in *CDKN2A* (i.e., rs3731249), causal variants at 2q22.3 and 9q21.31 loci may be tagged by other variants rather than rs17481869 and rs76925697, risk alleles of which are absent in East Asia. Therefore, we checked the linkage equilibrium (LD) block of these two SNPs in our cohort. No statistically significant signal was identified, suggesting racial specificity of these two loci. For *ETV6-RUNX1* subtype specific locus in *IGF2BP1*, although only 77 patients carried *ETV6-RUNX1* fusion, the risk allele frequency of rs10853104 has no obvious difference between patient and non-ALL controls (12.3% *vs.* 12.4%), suggesting that insignificance of this locus is probably induced by ethnic specificity rather than the small sample size. Moreover, similar trends were also observed for the rest of the insignificant variants, while enlarging sample size may increase the statistical power for evaluating the significance of some subgroup specific loci, such as *ERG* and *LHPP* (*P* = 0.07 and 0.08 in all pediatric patients).

For the GWAS approach, no novel locus has been identified, arguing for a larger sample size to identify potential novel susceptibility locus in Chinese patients in the future. However, we identified a potential novel locus (i.e., 15q25.3) that may have an impact on the susceptibility of all-age patients. Since we do not have an independent replication cohort, we checked the association of this locus in GWAS in previous reports with multi-ethnic populations to validate this signal [8]. Marginally significant was observed for the top signal at this locus (i.e., rs11638062) with *P* = 0.09. Interesting, another SNP at this locus (i.e., rs16977928 with *P* = 2.3 × 10^-5^ in our all-age GWAS of ALL susceptibility), which is in moderate LD with rs11638062 (r^2^ = 0.27, D’ = 0.66) in Caucasians, exhibits statistical significance in multi-ethnic population (*P* = 0.007). After considering ethnicity, despite the association trend in Caucasians and blacks, rs16977928 is only significant in Hispanics (*P* = 0.01), who are a mixture between Native American and Caucasians. Since the ancestors of Native Americans are considered to descend from East Asians [35], the causal variant for this locus may exhibit an ethnicity-specific manner in the East Asian population. Moreover, rs11638062 is located in the *AGBL1* gene, polymorphism in which was also associated with lung cancer risk in the Chinese population, suggesting its potential role on tumorigenesis [36]. On the other hand, rs73956024 is located in an enhancer region upstream of *HS6ST1* in B cells according to the public resource [37], and thus could be considered as a possible eQTL to possibly impact the expression level of the adjacent genes, including *HS6ST1*.

In the case of the impact of age on ALL, the difference of risk allele frequencies for the reported GWAS loci between patients and non-ALL controls decreased in adults compared with that in childhood except signals at *GATA3* locus, suggesting the majority of the known GWAS signals are age-specific for pediatric patients. Therefore, we conducted the first GWAS approach to screen ALL susceptibility locus in adult patients. rs3824662 at *GATA3* locus exhibits association at candidate level rather than genome-wide significance. On the other hand, a novel locus at 2q14.3 not only exhibits the most significant association with susceptibility in adult patients but also has the strongest impact on age at diagnosis, suggesting its potential role on age-specific leukemogenesis. Although no known gene located in the LD region of these SNPs, multiple ENCODE candidate cis-regulatory elements were identified, indicating the possible epigenetic effect of the causal variant in this region. Moreover, due to the limited samples and previous research on adult patients, validation for this locus is needed in independent cohorts with a large sample size in the future.

### Data availability

The datasets generated for this study can be found in Array Express in the Genome Variation Map (GVM) database (http://bigd.big.ac.cn/gvm) with the accession number of GVM000060.

### Ethics statement

This study was approved by the Ethics Committee of West China Second Hospital, Sichuan University, and informed consent was obtained from patients or their guardians, as appropriate.
